# Suppressive effects of sodium fluoride on cultured splenic lymphocyte proliferation in mice

**DOI:** 10.18632/oncotarget.11308

**Published:** 2016-08-16

**Authors:** Ping Kuang, Huidan Deng, Hengmin Cui, Lian Chen, Hongrui Guo, Jing Fang, Zhicai Zuo, Junliang Deng, Xun Wang, Ling Zhao

**Affiliations:** ^1^ College of Veterinary Medicine, Sichuan Agricultural University, Ya'an 625014, China; ^2^ Key Laboratory of Animal Diseases and Environmental Hazards of Sichuan Province, Ya'an 625014, China

**Keywords:** sodium fluoride, cytokine, cell cycle, splenic T lymphocytes, splenic B lymphocytes

## Abstract

Fluoride-induced immunotoxicity has been documented in vivo, but limited reports have focused on the effects of fluoride on lymphocytes in vitro. Therefore, we have examined the suppressive effects of sodium fluoride on cultured splenic lymphocytes in mice. CD3+ T lymphocytes, CD19+ B lymphocytes, cytokines, and cell-cycle markers were analyzed through the use of a cell-counting kit, western blot, and flow cytometery. Splenic lymphocytes were isolated from 3-week-old male ICR mice and exposed to sodium fluoride (0, 100, 500, and 1000 μmol/L) for 24 h. The percentages of CD3^+^, CD3^+^CD4^+^, CD3^+^CD8^+^ T lymphocytes and CD19^+^ B lymphocytes were decreased (P<0.05 or P<0.01) in the sodium fluoride-exposed cells. This finding was correlated with the alterations in expression levels of cytokine proteins and with evidence of cell-cycle arrest. Thus, protein expression levels of IL-2, TNF-α, IFN-γ, TGF-β were decreased (P<0.05 or P<0.01), and IL-10 protein expression levels were increased (P<0.05 or P<0.01). The percentage of lymphocyte in G1 phase was significantly increased (P<0.05 or P<0.01), while expression levels of cyclin E/D and CDK2/4 were markedly decreased (P<0.05 or P<0.01). These findings demonstrate that sodium fluoride exposure suppresses splenic lymphocyte proliferation, which is represented by reducing populations and activation of splenic T and B lymphocytes. Alterations of cytokine protein expression and cell cycle arrest are the molecular basis of the sodium fluoride-suppressed splenic lymphocyte proliferation, while reduction of T lymphocytes and B lymphocytes is the explanation of sodium fluoride-decreased splenic immune function in vitro.

## INTRODUCTION

Fluorine is an essential trace element for human health. With growing importance of fluorinated chemicals, fluorine-containing drugs are used in medicine as anesthetics, antibiotics, anti-cancer and anti-inflammatory agents, psychopharmaceuticals, and in many other applications [1]. However, long-term excessive intake of sodium fluoride (NaF) may result in serious skeletal and non-skeletal fluorosis [2–6] and dental fluorosis [7, 8]. Our previous studies documented fluorine-induced cytotoxicity, immunotoxicity, oxidative damage and pathological injury in the thymus [9], spleen [10–12], bursa of Fabricius [13], cecal tonsil [14–18], liver [19, 20], kidney [21–23], peripheral blood [24–27] and intestine [28–32] of broiler chickens. Recently, sodium fluoride toxicity in cultured cells, including lymphocytes, has been reported [4, 33–39].

The immune system protects the body against infections, diseases and cancers. The spleen, a peripheral organ of the body's immune system, maintains immune homeostasis [40, 41], and T and B lymphocytes are its principal components of immune reactions. Alterations of splenic lymphocytes in response to fluoride exposure can reflect fluoride immunotoxicity [42]. Cytokines are immunoregulatory proteins that are important host mediators for response to stress, infection and other forms of antigen invasion [43]. Cell cycle is central to maintaining homeostasis in multicellular organisms [44], and loss of cell cycle control may lead to imbalances in cell proliferation and to cell death, which contribute to various disease states, including tumor formation [44]. A close relationship exists between fluoride and these various immune systems and functions. However, studies of possible fluoride toxicity in immunologic components have mostly been limited to studies on splenic T lymphocytes, interleukin-2 (IL-2) and interferon gamma (IFN-γ) in vivo and in vitro; no systematic studies have been reported on the effects of fluoride toxicity on splenic T and B lymphocytes, and analysis of cytokine and cyclin protein expression and cell-cycle alteration.

Therefore, this study was conducted to investigate T and B lymphocyte proliferation; alteration of T and B lymphocyte populations; changes of protein expression levels of cytokines, including IL-2, IL-10, IFN-γ, transforming growth factor beta (TGF-β) and tumor necrosis factor alpha (TNF-α); and cell-cycle arrest in cultured murine splenic lymphocytes. The results provide new understanding of the molecular mechanism of the cytotoxic effect of NaF on splenic lymphocytes and immune function of the cultured splenic lymphocytes.

## RESULTS

### Half-maximal inhibitory concentration (IC50) of NaF on cultured splenic lymphocytes

**Figure 1:**
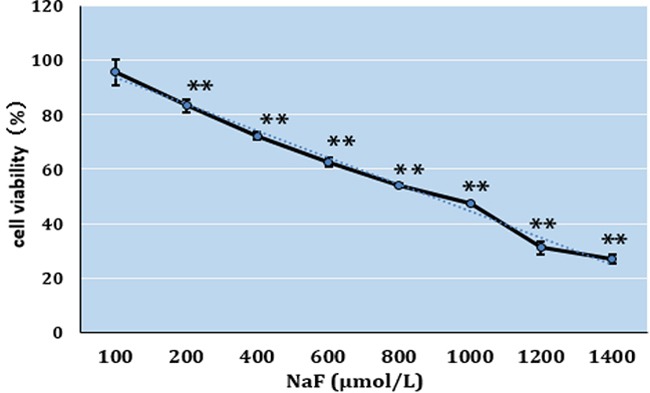
Cell viabilities of cultured splenic lymphocytes exposed to NaF at increasing concentrations as determined with cell counting kit-8

Figure 1 illustrated that the viability of splenic lymphocytes was decreased in response to increasing concentration of NaF at 24h. When the dose was over 1000 μmol/L, the cell activity was under 50%. By calculating the IC50 in this study was 1000 μmol/L. Based on IC50, doses of 100, 500, and 1000 μmol/L NaF were chosen for study of lymphocyte proliferation.

### Effect of NaF treatment on proliferation of cultured splenic lymphocytes

**Figure 2:**
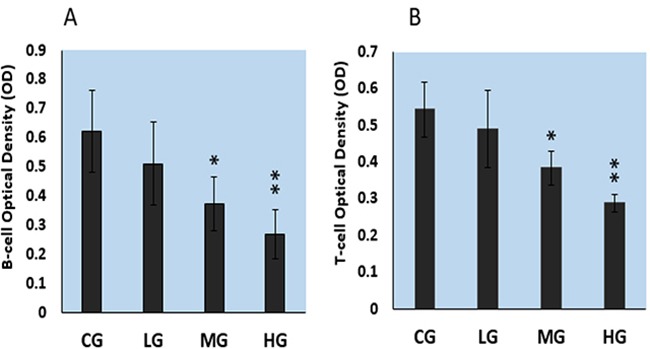
The effects of NaF on cell proliferation of splenic T and B lymphocytes as determined with cell counting kit-8 **A.** Splenic B lymphocyte proliferation by NaF combined with LPS stimulation. **B.** Splenic T lymphocyte proliferation by NaF combined with ConA stimulation. CG: control group, LG: low-dose NaF group, MG: middle-dose NaF group and HG: high-dose NaF group, LPS: Lipopolysaccharide, ConA: Concanavalin A * P < 0.05, compared with the control group; ** P < 0.01, compared with the control group.

As illustrated in Figure 2A-2B, the proliferation of splenic B lymphocytes, stimulated with lipopolysaccharide, and of T lymphocytes, stimulated with concanavalin A, was decreased in a dose-dependent manner with increasing concentration of NaF. Lymphocyte proliferation was significantly lower in the middle-dose (MG) (P<0.05) and high-dose (HG) (P<0.01) NaF groups than in the control group (CG).

### Effect of NaF treatment on cultured CD3^+^, CD3^+^CD4^+^, and CD3^+^CD8^+^ splenic T lymphocytes and CD19^+^ splenic B lymphocytes

**Figure 3:**
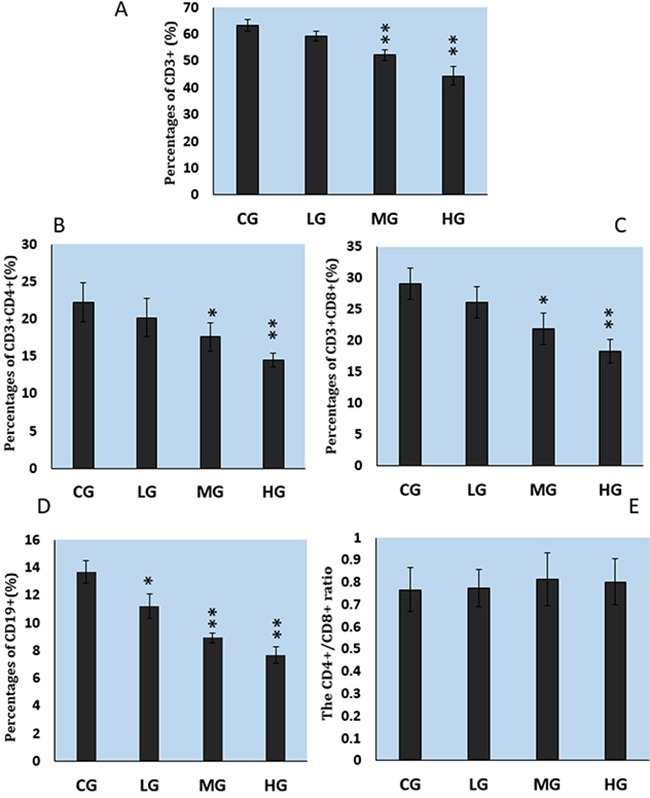
Changes of cultured splenic lymphocytes as determined with flow cytometry **A-C.** CD3^+^, CD3^+^CD4^+^, and CD3^+^CD8^+^ T lymphocyte percentages. **D.** CD19^+^ B lymphocyte percentages. **E.** CD4^+^/CD8^+^ ratio. CG: control group, LG: low-dose NaF group, MG: middle-dose NaF group and HG: high-dose NaF group. * P < 0.05, compared with the control group; ** P < 0.01, compared with the control group.

As illustrated in Figure 3A-3C, the percentages of CD3^+^, CD3^+^CD4^+^, and CD3^+^CD8^+^ T lymphocytes were significantly lower in the MG (P<0.05) and HG (P<0.01) than in the CG. Similarly, the percentages of CD19^+^ B lymphocytes were lower in the LG (P<0.05) and MG and HG (P<0.01) than in the CG (Figure 3D). However, there were no significant changes in the CD4^+^/CD8^+^ ratios (Figure 3E).

### Effect of NaF treatment on cytokine protein expression in cultured splenic lymphocytes

**Figure 4:**
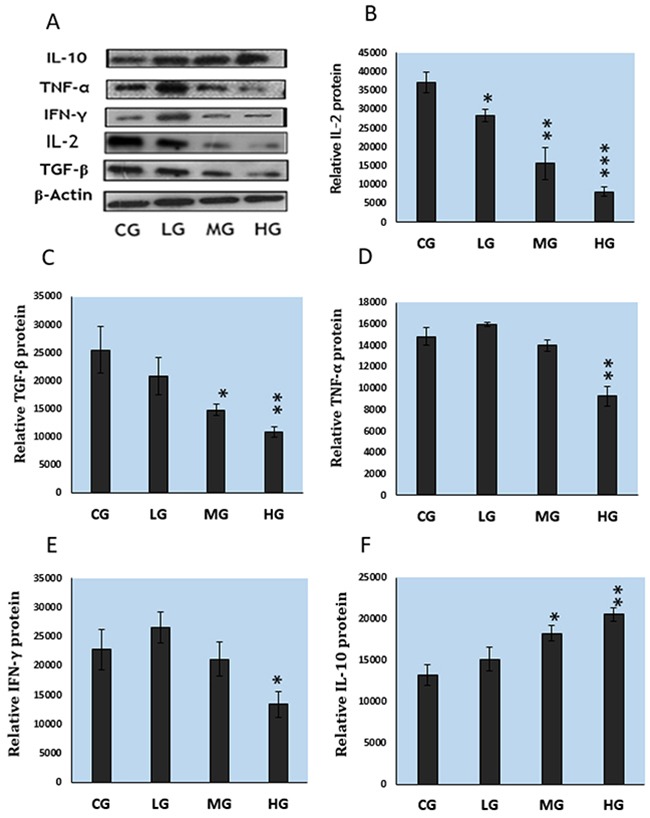
Protein expression levels of cytokines in cultured splenic lymphocytes **A.** Western blot assay. **B-F.** Quantitative measurement of the relative protein expression of IL-2, TGF-β, TNF-α, IFN-γ, and IL-10 CG: control group, LG: low-dose NaF group, MG: middle-dose NaF group and HG: high-dose NaF group.* P < 0.05, compared with the control group; ** P < 0.01, compared with the control group.

As shown in Figure 4A-4F, NaF decreased protein expression levels of IL-2, TGF-β, IFN-γ, and TNF-α, and increased protein expression levels of IL-10 in cultured splenic lymphocytes.

### Effect of NaF treatment on cell cycle, and cyclins and cdks protein expression in cultured splenic lymphocytes

**Figure 5:**
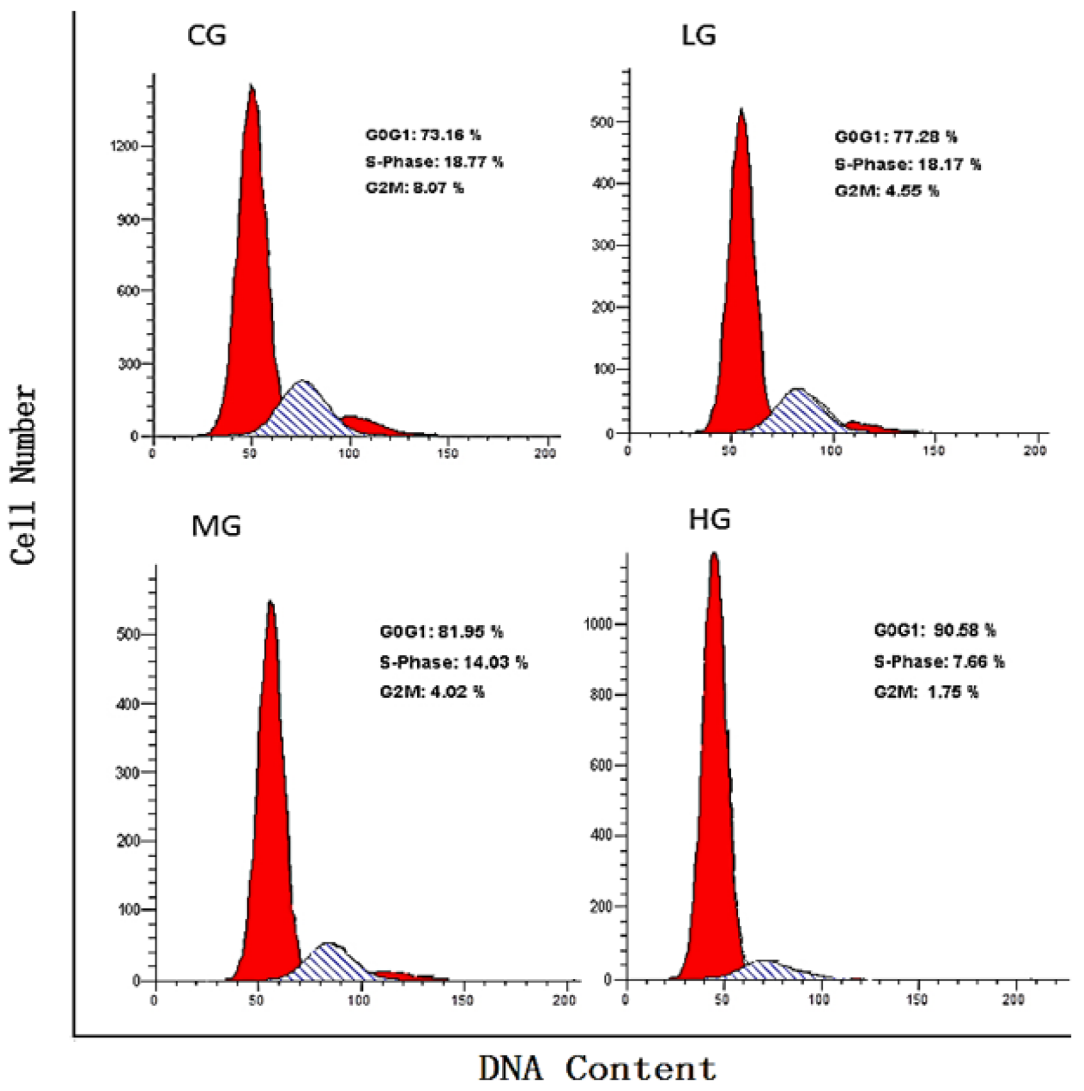
Effects of NaF on the DNA content of splenic cells in various phases of the cell cycle, as measured with flow cytometry CG: control group, LG: low-dose NaF group, MG: middle-dose NaF group and HG: high-dose NaF group.

**Figure 6:**
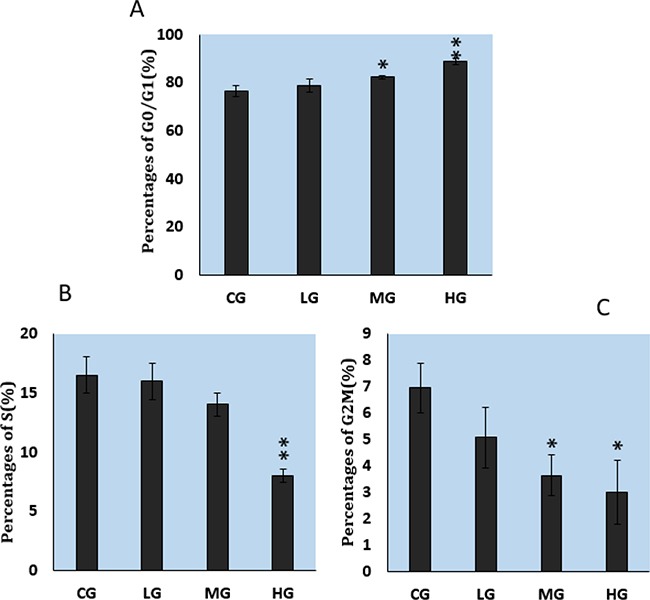
Effects of NaF on percentages of cultured splenic lymphocytes in various stages of the cell cycle, as measured with flow cytometry **A.** G0/G1 phase. **B.** S phase. **C.** G2M phase. CG: control group, LG: low-dose NaF group, MG: middle-dose NaF group and HG: high-dose NaF group. * P < 0.05, compared with the control group; ** P < 0.01, compared with the control group.

As illustrated in Figure 5, NaF inhibited DNA synthesis of cultured splenic lymphocytes in a dose-dependent manner. Figure 6 illustrates that NaF treatment, at MG and HG, increased the cell percentage of cells in the G0G1 phase and decreased the percentage in the S and G2M phases.

**Figure 7:**
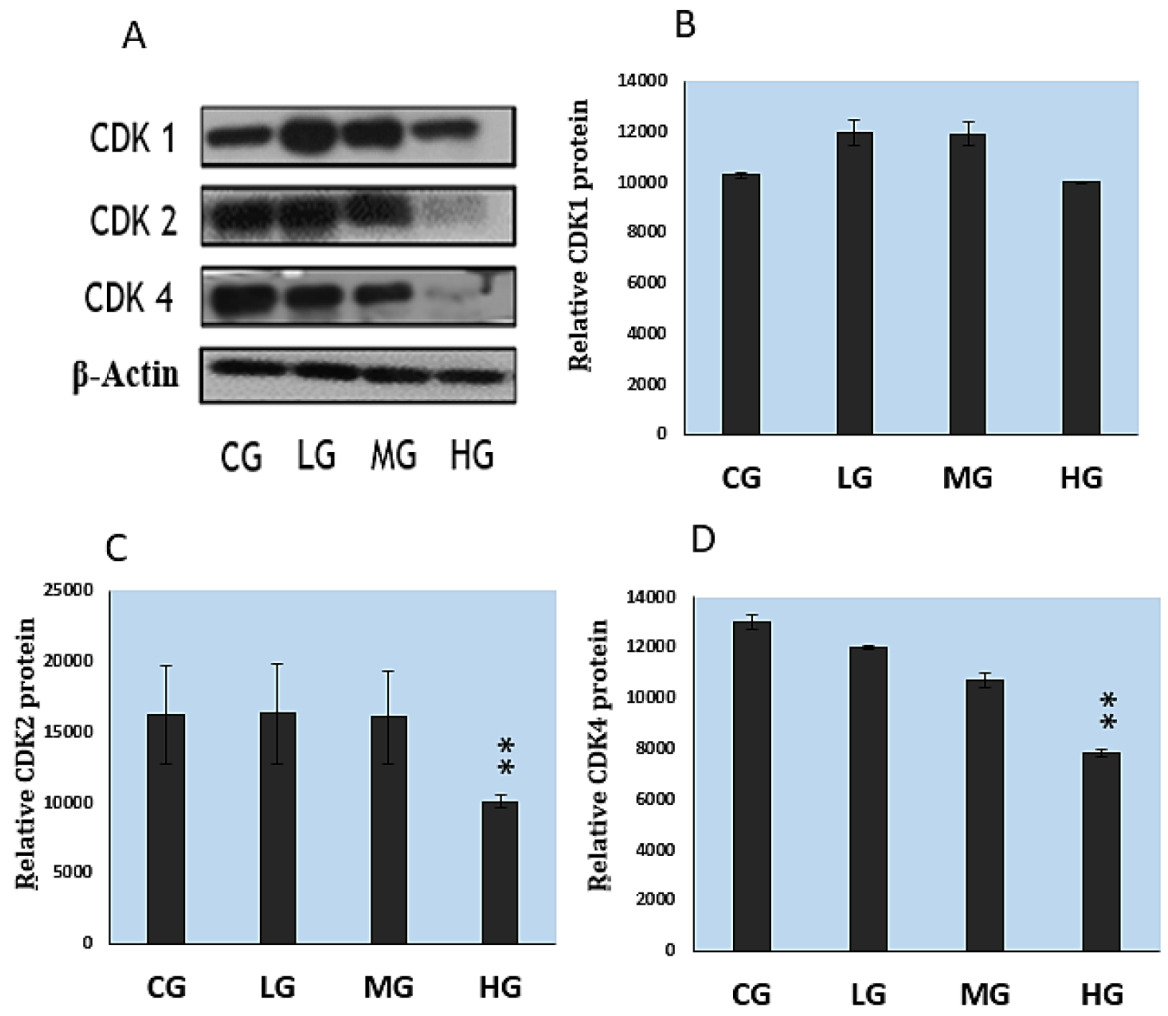
Protein expression levels of cyclin-dependent kinases in cultured splenic lymphocytes, as determined with quantitative measurement of western blots **A.** Western blot assay. **B-D.** Quantitative analysis of CDKs relative protein expression. CG: control group, LG: low-dose NaF group, MG: middle-dose NaF group and HG: high-dose NaF group. * P < 0.05, compared with the control group; ** P < 0.01, compared with the control group.

**Figure 8:**
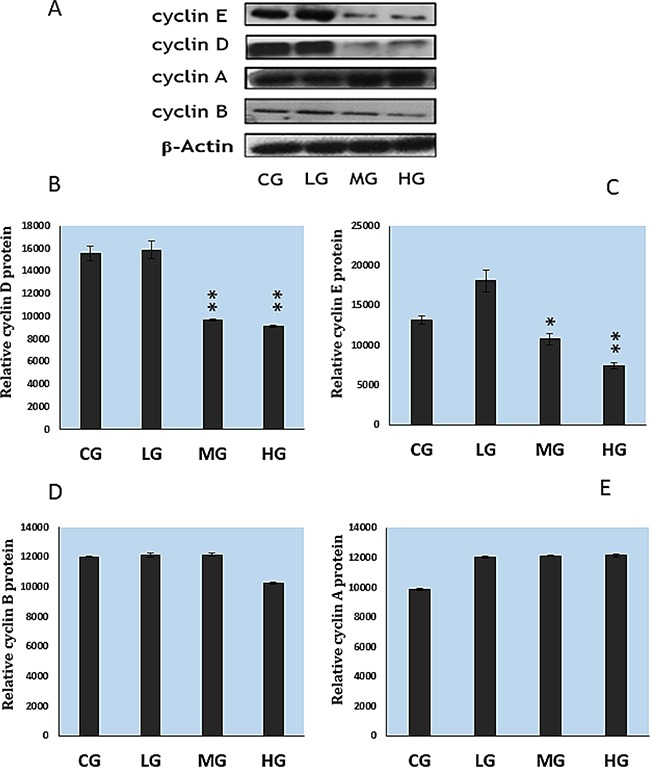
Cyclins expression in cultured splenic lymphocytes as determined with western blot and quantitative measurement **A.** Western blot show. **B-E.** Quantitative analysis of cyclins relative protein expression. CG: control group, LG: low-dose NaF group, MG: middle-dose NaF group and HG: high-dose NaF group. * P < 0.05, compared with the control group; ** P < 0.01, compared with the control group.

To reveal the mechanism of NaF-arrested G1 phase of the cell cycle, we determined expression levels of the G1 phase-related regulatory molecules, cyclins and cdks, with western blot. As illustrated in Figure 7A-7D, NaF treatment at the high dose (HG) significantly (P<0.01) decreased the protein levels of CDK2 and CDK4 but had no effect on CDK1. In Figure 8A-8E, it can be seen that NaF treatment at the MG and HG concentrations significantly decreased cyclins D and E (P<0.05-P<0.01), whereas it had no effect on cyclins A and B (P>0.05).

## DISCUSSION

There are no systematic studies on the effects of fluoride toxicity on splenic T and B lymphocytes, and analysis of cytokine and cyclin protein expression and cell-cycle alteration at present. Thus, this study focused on the cultured splenic lymphocyte proliferation at 24h, and aimed to provide new experimental evidences for understanding the molecular mechanism of NaF cytotoxicity in the future studies. Indeed, we found in this in vitro study that NaF suppressed the proliferation of cultured splenic lymphocyte by reducing T and B lymphocyte populations and viability (Figure 1, 2); altering protein expression of cytokines IL-2, IL-10, IFN-γ, TGF-β and TNF-α; and arresting the cell cycle. It is firstly observed that NaF reduces splenic B lymphocytes and alters splenic lymphocyte TGF-β and IL-10 protein expression in vitro, and arrests splenic lymphocyte cell cycle in vitro.

Splenic lymphocytes play very important roles in body's immune response, which are mainly involved in cellular immunity and humoral immunity [40]. Thus, in this study, we chose splenic T and B lymphocytes for evaluation of the immune status of mice after NaF exposure. Treatment with NaF directly decreased the proliferation of splenic T and B lymphocytes--CD3^+^ T lymphocytes, CD3^+^CD4^+^ T lymphocytes, CD3^+^CD8^+^ T lymphocytes and CD19^+^ B lymphocytes (Figure 3). This finding is consistent with the results of Liu et al [15, 16], and Luo et al [29], who found that dietary high fluorine reduced the numbers of T lymphocytes and B lymphocytes in the cecal tonsil, and B lymphocytes in the intestinal mucous [32] of broiler chickens. Also, Peng et al.[38] has reported that fluorine ion decreases cultured splenic CD3^+^ T lymphocytes of male Kunming mice in vitro. Accordingly, the reduction of T lymphocytes and B lymphocytes seems to account for NaF-decreased splenic immune function.

Cytokines are critical regulators of the immune system by activating and modulating the function of immune cells [45]. Thus, there is a close relationship between lymphocytes and cytokines in the spleen. In this in vitro study, NaF altered the protein expressions of IL-2, TNF-α, IFN-γ, IL-10 and TGF-β in cultured splenic lymphocytes (Figure 4). IL-2, TNF-α, and IFN-γ are crucial cytokines in the regulation of immune function, through enhanced cell-mediated immunity. In this study, protein expression levels of IL-2, TNF-α, and IFN-γ were decreased (Figure 4B, 4D, 4E) by NaF, indicating that NaF could reduce the population of T lymphocytes and likely result in impaired cell-mediated immune function. The reduction of IL-2, TNF-α and IFN-γ expression was consistent with previous reports in cecal tonsil and intestine of broiler chickens fed high fluoride diets [19, 35]. Also, IL-2 mRNA expression levels have been reduced in cultured splenic T lymphocyte in the presence of NaF [39].

IL-10 and TGF-β are inhibitory cytokines, synthesized by T cells and B cells [46], and IL-10 combined with TGF-β can promote the production of IgA [47]. To our knowledge, there have been no reports on NaF-altered IL-10 and TGF-β protein expression in splenic lymphocyte in vivo or in vitro. In this study, TGF-β protein expression levels were decreased and IL-10 protein expression levels were increased in NaF-treated cells (Figure 4C, 4F). The alteration of these expression levels indicates that NaF exposure reduces populations of T lymphocytes and B lymphocytes, which further impacts splenic immune function in mice.

The suppression of splenic lymphocyte proliferation is closely correlated with the cell cycle in addition to the relationship between suppression of splenic lymphocyte proliferation and cytokines. Our results showed that NaF caused G1 phase cell-cycle arrest (Figure 5–6), a result that is consistent with reports on fluoride-induced increases in the proportion of cells in the G0/G1 phase in the spleen [12, 48]. Progression from G1 to S phase of the mammalian cell cycle is regulated by cyclin D-dependent kinases, including Cdk4 and Cdk6, binding to D-type cyclins, and by Cdk2 binding to cyclins E or A [49, 50]. In order to define NaF-induced G1 phase cell-cycle arrest in the cultured splenic lymphocytes, we measured cell-cycle regulatory proteins. We found that protein expression levels of cyclin E, cyclin D, CDK2 and CDK4 were significantly reduced in the MG and HG (Figure 7C-7D; Figure 8B-8C), while protein expression levels of cyclin B, cyclin A, and CDK1 were not changed. Ngoc et al.[51] have reported that NaF can reduce cyclin E levels in mouse embryonic stem cells, indicating that NaF treatment slows the G1 process and blocks the G1/S transition.

We conclude that NaF in the 500, 1000 μmol/L suppresses cultured lymphocyte proliferation, which is represented by reducing population and activation of splenic T and B lymphocytes. Cytokine protein expression alteration and cell cycle arrest are the molecular basis of NaF-suppressed splenic lymphocyte proliferation. Also, we believe this in vitro study is the first to show sodium fluoride-decreased splenic immune function by reducing not only T lymphocytes, but also B lymphocytes.

## MATERIALS AND METHODS

### Animals

Three-week-old healthy male ICR mice were provided by the Experimental Animal Corporation of DOSSY at Chengdu, China.

Our experiments involving the use of mice and all experimental procedures were approved by the Animal Care and Use Committee, Sichuan Agricultural University.

### Lymphocyte isolation, culture and treatment

Mice were anesthetized and euthanized, then soaked in 75% ethanol for 3 to 5 min. The spleens were removed at laparotomy, washed with cold phosphate-buffered saline (PBS, pH 7.4), and placed in a 200-mesh stain steel sieve over a culture dish containing 4-5 mL lymphocyte isolation separation medium. The spleens were ground into small pieces with the plunger of glass syringe. The liquid was transferred into a centrifuge tube and overlaid onto a layer of 200-500 μL RPMI–1640 and centrifuged at 800×*g* for 30 min at room temperature, with three layers were formed. The middle, milky layer containing lymphocytes was transferred into a test tube. The lymphocytes were washed twice with PBS and suspended in RPMI-1640 medium with 10% fetal calf serum and transferred into a culture bottle. The mentioned procedures were performed under sterile condition. The viability of the lymphocyte was estimated according to trypan blue exclusion criteria.

To analyze T and B lymphocytes, lymphocyte proliferation, cytokines, cell cycle by cell counting Kit-8 (CCK-8, Beyotime, Jiangsu, China), western blot and flow cytometry. The splenic lymphocytes were cultured in the RPMI-1640 medium (supplemented with 10% fetal calf serum, 100 μ/mL penicillin, and 100 μg/mL streptomycin), containing 0 (control group, CG), 100((low-dose group, LG), 500 (medial-dose group, MG), and 1000 (high-dose group, HG) μmol/L NaF. Experiments were repeated in treplicate. The cells were maintained in a humidified incubator for 24 h at 37°C with 5% CO2.

### Cell viability assay

1×10^5^ splenic lymphocytes were seeded in 96-well cell culture plates and were exposed to NaF (0, 100, 200, 400, 600, 800, 1000, 1200, 1400 μmol/L) for 24 h with 5% CO_2_. The cells were then incubated with 10μL CCK-8 agents (Beyotime, Jiangsu, China) in 100μL medium for 4 h at 37°C. The optical density (OD) was measured by using the Thermo Scientific™ MμLtiskan microplate spectrophotometer (Thermo Fisher Scientific; Waltham, MA, USA) at a wavelength of 450 nm.

### Cell proliferation assay

Splenic lymphocytes were seeded into 96-well cell culture plates (1×10^5^/well) and exposed to NaF (0, 100, 500, and 1000 μmol/L), and lipopolysaccharide (LPS, 10 μg/mL) or concanavalin A (ConA, 5 ug/mL) was added. The cells were incubated at 37°C under 5% CO_2_ for 24 h. At the indicated time points, 10 μL CCK-8 solution was added to each well, and the plates were incubated for 4 h. The optical density (OD) was measured at 450 nm.

### Protein expression analysis by western blot

Cells were lysed, and proteins were extracted with RIPA lysis buffer and kept in Laemmli buffer. Protein samples were resolved on SDS-PAGE (10%–15% gels) and transferred to nitrocellulose filter membranes. Membranes were blocked with 5% fat-free milk for 1 h and incubated with primary antibodies overnight at 4°C. The primary antibodies were cyclin D/E/B/A, CDK1/2/4 (Abcam, UK) and TNF-α, IFN-γ, TGF-β, and IL-2, IL-10 (Santa Cruz, USA). The membranes were then washed with PBS-tween and incubated with biotin-conjugated secondary antibodies (Santa cruz, USA) for 1 h, and washed again with PBS-tween. Blots were visualized by ECL^TM^ (Bio-Rad, Hercules, CA, USA) and X-ray film.

### Lymphocyte and cell cycle analysis by flow cytometer

The percentages of cultured splenic lymphocyte subsets were measured by flow cytometer, using monoclonal fluorescein isothiocyanate (FITC) anti-mouse CD3^+^, phycoerythrin (PE) anti-mouse CD4^+^, PerCp anti-mouse CD8^+^ and FITC anti-mouse CD19^+^ (BD Biosciences, San Jose, CA, USA) as monoclonal antibodies labeled, respectively. The results were analyzed using the Cell Quest computer program.

The cell cycle was measured by flow cytometer. Cells were incubated for 30 min at room temperature in the dark with 0.15% Triton X-100 and propidium iodide (PI). The results were analyzed by the use of the Mod Fit LT for Mac V3.0 computer program.

### Statistical analysis

Data were expressed as mean±standard deviation (SD). One-way analysis of variance (ANOVA) procedure in SPSS 17.0 software was used to assess statistical significances between F-treated group and control group. A value of P<0.05 was considered significant, and P<0.01 was markedly significant.
